# A systematic review of the association between coping strategies and quality of life among caregivers of children with chronic illness and/or disability

**DOI:** 10.1186/s12887-019-1587-3

**Published:** 2019-07-01

**Authors:** Alana Fairfax, Jamie Brehaut, Ian Colman, Lindsey Sikora, Alessia Kazakova, Pranesh Chakraborty, Beth K. Potter, in collaboration with the Canadian Inherited Metabolic Diseases Research Network

**Affiliations:** 1 School of Epidemiology and Public Health, Faculty of Medicine, University of Ottawa, 600 Peter Morand Drive, Ottawa, ON Canada; 2 Ottawa Hospital Research Institute, Ottawa, ON Canada; 3 Health Sciences Library, University of Ottawa, Ottawa, ON Canada; 4 Children’s Hospital of Eastern Ontario, Ottawa, ON Canada

**Keywords:** Caregiver health, Caregiving complexity, Quality of life, Coping, Systematic review

## Abstract

**Background:**

Parents of children with chronic illness have reported decreased psychological and physical quality of life (QoL) relative to parents of children without such illness, which may be associated with the extent of complexity involved in the caregiving role. Given that coping strategies have been reported to influence QoL, our goal was to synthesize existing research about the association between coping strategies and QoL in caregivers of children with chronic illness. We were particularly interested in whether coping strategies may mediate the association between caregiving complexity and QoL, or may modify the association.

**Methods:**

We developed an electronic search strategy to identify relevant citations in Medline, EMBASE, PsycINFO and CINAHL. Two reviewers independently assessed retrieved citations against pre-specified inclusion criteria in two stages of screening. One reviewer abstracted data on study characteristics, methods to address confounding, measurement tools, risk of bias, and results with respect to associations of interest. A second reviewer validated extracted data. We summarized results narratively.

**Results:**

2602 citations were screened and 185 full-text articles reviewed. The 11 articles that met inclusion criteria addressed 5 diseases and included a total of 2155 caregivers. Ten of the 11 included studies were cross-sectional. We identified some evidence that coping was associated with QoL: in three studies, coping strategies considered to be adaptive were positively associated with psychological QoL while in one study, maladaptive strategies were negatively associated with psychological QoL. Only two studies considered coping as a potential mediating variable in the association between caregiving complexity and parental QoL, with inconsistent findings and challenges in interpreting cross-sectional associations. No studies considered coping as a moderating variable. The variability among instruments used to measure key constructs, particularly coping strategies, made it difficult to synthesize results.

**Conclusions:**

We found that coping strategies may be associated with psychological QoL among parents of children with chronic illness. We also identified important research gaps related to the consistent and clear measurement of coping strategies and their prospective association with QoL. Understanding how coping strategies are associated with QoL is important to inform the development of interventions to support families of children with chronic illness.

**Electronic supplementary material:**

The online version of this article (10.1186/s12887-019-1587-3) contains supplementary material, which is available to authorized users.

## Background

Pediatric chronic illness affects not only the child but the entire family [1, 2]. In Canada, the setting for this study, 3.7% of children 15 years or younger were reported to have a disability [3] and 500,000 children were estimated to have a long-term chronic illness or mental illness [4]. Among children with chronic illness, nearly half experience severe disease and 8% experience ongoing activity limitations [4–6]. The number of children with diagnosed disability is likely to increase over time as children with chronic illness are living longer and healthier lives [4–6]. For example, many children diagnosed with illnesses that were once considered severely life-limiting, such as cystic fibrosis or muscular dystrophies, are now living into adulthood [7, 8]. Care pathways for children with severe illness have gradually shifted away from hospital-based care and toward home-based care, often coordinated by parents and families [8]. Parents of children with chronic illness are thus not only primary nurturers in their children’s lives but also key members of their children’s health care teams [8].

Parents of children with chronic illness can experience increased caregiver challenges relative to parents of children without such needs, including increased medical related costs, challenges with child care, and constrained employment opportunities [5, 9–14]. These challenges, combined with direct parental responsibilities in health care, may increase the impact of caregiving on parents of children with chronic illnesses [15]. Evidence suggests that the challenges experienced by caregivers of children with chronic illness may influence overall caregiver health [12, 16–20]. For example, a Canadian study found that caregivers of children with chronic health problems had more than twice the odds of having symptoms of depression, physical limitations, and chronic health problems of their own compared to caregivers of healthy children [21]. When the health of caregivers of children with chronic health problems was examined over 10 years, poorer self-reported caregiver health was associated with child health needs for the entirety of the 10 years [15]. Additionally, caregivers of children with severe health problems reported worse general health than caregivers of children with less severe health problems, who, in turn, reported worse health compared with caregivers of healthy children [15]. Findings from these studies support the idea that caregiver health is associated with the complexity of the caregiving role [15]. Caregiving complexity for parents of children with chronic illness may be viewed as a multi-faceted concept that incorporates the impact of the clinical or medical severity of the child’s disease as well as the social, time, and economic implications of caregiving, which may vary according to child, parent, family, and environmental circumstances [22].

Factors that affect the well-being of parents or caregivers of children with chronic illness are not well documented [23]. One area of study has been the potential association of coping strategies with caregiver well-being [24]. A challenge to understanding how coping may relate to the well-being of caregivers of children with chronic illnesses is that there is not a consensus in the literature regarding how coping is conceptualized and measured [25–27]. Coping strategies are thought to be context dependent, indicating that both the stressor and the environment in which the stressor is presented contribute to the coping strategy used [28]. However, habitual coping strategies are also believed to develop and these differ among families [28]. Most instruments designed to measure coping responses classify these into specific strategies, approaches, or styles of coping using subscales [29–33]. However, their classification systems differ. For example, some authors distinguish between adaptive or positive coping strategies (e.g., maintaining social connections) and maladaptive or negative strategies (e.g., substance use) [34, 35]; some distinguish between problem-focused coping (e.g., planning or problem-solving) and emotion-focused coping (e.g., escape-avoidance) [36]; and some describe different approaches to coping that would nevertheless all be considered adaptive, in that higher scores on any subscale within a measurement instrument would be interpreted as positive in terms of managing stress [37]. These classification systems imply different overall conceptions of how people cope with stressful situations and, in some cases, different assertions about which approaches may be assumed to be “better” with respect to successful stress management and well-being. These differences in measurement also render it challenging to make comparisons across studies.

Our goal was to synthesize existing research about the association between coping strategies and quality of life (QoL) in caregivers of children with chronic illness or disability. Based on Carona et al. [24], Dardas and Ahmad [23], and Lyons et al. [38], we were particularly interested in whether coping strategies may moderate the association between caregiving complexity and QoL (i.e., whether parental use of coping strategies would modify the influence of caregiving complexity on QoL, for example by mitigating the stress associated with caregiving), and/or whether coping strategies would mediate the association between caregiving complexity and QoL (i.e., whether coping strategies would be an intermediate variable that helps to explain the association between caregiving complexity and QoL). It has been argued that because different childhood illnesses or disabilities may have similar social and psychological impacts on caregivers and families, a ‘non-categorical’ approach (i.e., one that does not focus on a specific diagnosis but rather on a set of common challenges) is best when studying such impacts [39–41]. To align with this recommendation, we chose to focus on chronic pediatric illnesses that would be likely to be diagnosed early in childhood, have important impacts on caregivers with respect to the need for chronic or long-lasting home management, and require ongoing pediatric specialist care (i.e., medical, surgical, and/or nutritional intervention). To the best of our knowledge this is the first systematic review to consider the association between coping strategies and caregiver QoL in this population.

## Methods

### Protocol and registration

The protocol for this review was written following the PRISMA-P (Preferred Reporting Items for Systematic Reviews and Meta-Analyses- Protocol) checklist [42]. The protocol is registered with PROSPERO under the following registration number: CRD42017069316.

### Eligibility criteria, search strategies and screening

The eligibility criteria for articles to be considered in this review are described in Table 1. Because of the non-specific nature of the search terms in this field, for feasibility, it became necessary to narrow the search strategy. Because we were interested in diseases that would present similar challenges with respect to caregiving, as described above, we chose to focus on chronic pediatric illnesses with the following characteristics: (1) the disease would likely be diagnosed early in childhood and would have long-term intense home management requirements with an important impact on caregivers (to operationalize this, we focused on diseases with long-lasting manifestations and with genetic, metabolic, and/or neurologic etiology) and (2) the disease would require care from pediatric specialists involving nutritional, surgical and/or medical intervention.

**Table 1:** Eligibility criteria for systematic review of the association between coping strategies and quality of life among caregivers of children with chronic illness and/or disability

Population	Parents/guardians of children (≤18 years of age) with a chronic illness or disability for which the etiology/manifestations are genetic, metabolic or neurologic and specialist pediatric care is required, involving surgical, medical and/or nutritional intervention
Intervention or exposure	Coping (with or without caregiving complexity)
Comparator	Quantitative comparison of QoL (outcome) among individuals using different coping strategies or using strategies to different degrees
Outcome	QoL
Study characteristics	Peer reviewed, English-language, full text article describing primary study that includes ≥5 participants

With a health sciences librarian (LS), we developed a search strategy to identify eligible studies from the following electronic databases: Medline (via Ovid), EMBASE (via Ovid), PsycINFO (via Ovid), and CINAHL (via EBSCOHost) (search strategy, Additional file 1). A combination of Medical Subject Headings (MeSH) and text words focused on the following concepts: coping strategies, QoL, and childhood diseases that were of interest to our study aims. Given the broad range of childhood diseases of interest, we searched for relevant etiology using MeSH only, while the remaining concepts were searched using both MeSH and text words. The requirements for childhood diseases requiring care from pediatric specialists involving nutritional, surgical and/or medical intervention, and the notion of a long-lasting pediatric condition were adjudicated at the screening stage. The search was limited to children under the age of 18 and only English-language articles were considered. The initial search was conducted on December 2, 2016; the search was updated on March 29, 2019.

To identify additional eligible articles that may have been missed by the search of electronic databases described above, the citation list and bibliographies of relevant articles were also searched for applicable studies. We completed a grey literature search in clinicaltrials.gov, the Global Rare Diseases (Patient) Registry and Data Repository, Health Canada’s Trial Registries, World Health Organization (WHO) registry, and Google scholar. A combination of key text words that formed the database search strategies was used to complete the search of grey literature.

In the first phase of screening, two reviewers independently screened returned titles and abstracts using pre-specified eligibility criteria (Table 1). Any discrepancies between reviewers were discussed; if there was remaining uncertainty of a citation’s relevance, it was retained and further considered in the second phase of screening. In the second phase, full-text manuscripts of citations deemed relevant during the first screening phase were independently screened by two reviewers. All discrepancies were resolved through discussion, with third party consultation when needed. The number of articles excluded during each screening phase as well as reasons for study exclusion during the second phase were described using a flow diagram following the PRISMA statement [43]. Cohen’s kappa was used to measure inter-rater reliability during the second screening phase, with the following formula:

$$ k=\frac{\Pr \left(\mathrm{a}\right)-\Pr \left(\mathrm{e}\right)}{1-\Pr \left(\mathrm{e}\right)} $$

where Pr(a) is the actual observed agreement between the reviewers and Pr(e) represents the chance agreement between the reviewers [44].

When screening against eligibility criteria (Table 1), to distinguish QoL from other related aspects of caregiver health, QoL was considered to have been measured if studies: 1) examined both physical and psychological caregiver health; and 2) used a QoL measurement instrument. If only psychological well-being or physical well-being was assessed then the study was considered ineligible. Coping was considered to have been measured if the author/s 1) used a coping instrument that measured coping strategies used by the parent caregiver or 2) considered coping strategies to be measured in the parent. Caregiving complexity did not have to be measured for a study to be considered relevant for the review but was examined in association with QoL if measured.

### Data extraction and analysis

Two reviewers piloted the data extraction form and then one reviewer extracted the data from relevant articles and another reviewer verified the extracted data. We abstracted information on study characteristics, measures used to assess coping strategies, QoL, and caregiving complexity, as well as methods and results for relevant analyses used to assess the relationships among these variables. Due to clinical heterogeneity among diseases that were the focus of relevant studies and anticipated methodologic heterogeneity in concepts, measures, and analyses, it was decided, a priori, to complete a narrative synthesis rather than to quantitatively synthesize the findings. We narratively synthesized the following information: study characteristics and instruments used to measure key constructs; study quality (see below); association between caregiving complexity and QoL among parents of children with chronic illness; association between coping and QoL; and, role of coping as a mediator of the association between caregiving complexity and QoL or as a moderator of that association.

### Study quality assessment

The methodological quality of included studies was examined using the 14-item Quality Assessment Tool for Observational Cohort and Cross-sectional Studies from the National Heart, Lung and Blood Institute [45], which yields a final quality rating of good, fair, or poor for each included study. Only one study in the final sample was a randomized controlled trial (RCT) and since the analyses of interest were not separated based on intervention group, we treated that study as observational for the purposes of quality appraisal in order to use the same appraisal tool across all studies. The quality of the RCT was also secondarily assessed using the Cochrane risk of bias tool for RCTs [46]. One reviewer critically appraised study quality and a second reviewer verified the assessments.

## Results

### Search and screening

**Fig. 1:**
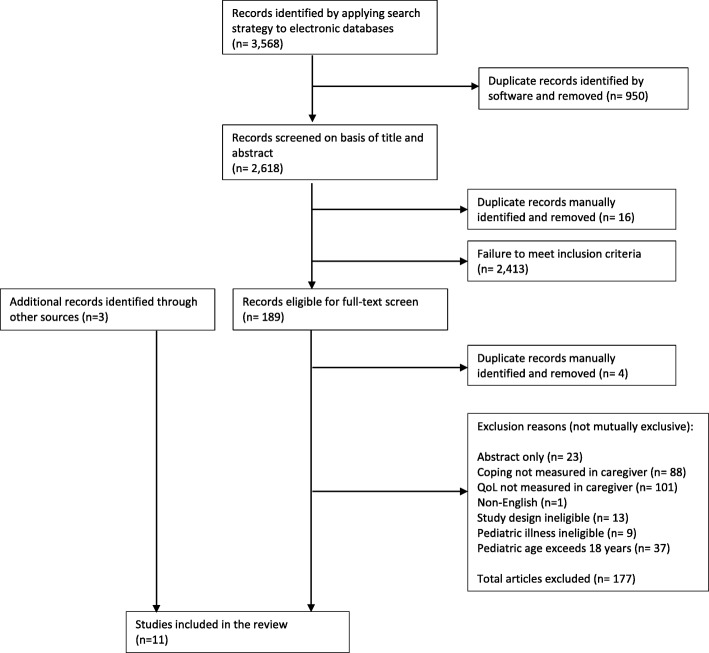
PRISMA flow diagram for the systematic review of the association between coping strategies and quality of life among caregivers of children with chronic illness and/or disability

The Medline (1556), Embase (1341), PsycINFO (197), and CINAHL (474) searches yielded a total of 3568 citations. Citations were imported into the review manager program, Covidence, where 950 duplicates were removed. An additional 16 duplicates were manually identified during the first phase of screening, yielding 2602 unique citations from the initial search (Fig. 1). Following the first screening phase, 189 citations were identified for full-text review, of which four were identified as duplicates. During the second screening phase 177 articles were excluded. Cohen’s kappa during the second screening phase was calculated to be 0.8, which indicates a strong level of agreement between the reviewers [44]. The majority of excluded studies satisfied more than one reason for exclusion (Fig. 1): 9 studies described pediatric illnesses that were deemed ineligible for this review; 37 articles included populations that exceeded 18 years of age and did not report results separately for children aged 18 years and younger; one study was non-English; and 23 were abstracts only. A large number of excluded studies (101 studies) did not measure overall QoL in caregivers but most often measured only psychological well-being. Another large quantity (88 studies) did not satisfy the coping eligibility criteria and 13 articles had ineligible study designs (e.g., commentary, descriptive analysis only without examining the association between coping and QoL). The grey literature search yielded one relevant article. Two additional articles were manually identified through reference list searches of relevant studies. A total of 11 articles were included in the review for further analysis.

### Characteristics of the included studies

The 11 included studies considered 5 diseases (Table 2, one study included both cerebral palsy and epilepsy): autism (3 studies), cerebral palsy (4 studies), diabetes (2 studies), epilepsy (2 studies), and hemophilia (1 study). Ten of the 11 included articles were cross-sectional, with one study being an RCT. Four studies were conducted in the USA while the remaining 7 studies varied geographically, representing Canada, Portugal, Spain, Jordan, Israel, Iran, and Taiwan. A total of 2155 caregivers were included in the review. In nearly all of the included studies, the vast majority of study respondents were mothers (Table 2).

**Table 2:** Study characteristics of included studies

Author, year (country)	Study design	Sample size	Illness	Inclusion criteria	Caregiver relation to child	Mean age of caregivers (SD or range)	Age of children
Carona et al., 2014 [24] (Portugal)	Cross-sectional	156 (epilepsy, *n* = 65; cerebral palsy, *n* = 91)	Epilepsy and cerebral palsy	Parent of a child aged 8–18 years who had been diagnosed with epilepsy or cerebral palsy by a physician, and assumed the primary caregiving role at the time of assessment.	Mother (87.7% epilepsy; 90.1% cerebral palsy)	42.42 years (7.20) epilepsy; 41.47 years (6.26) cerebral palsy	12.52 years (2.88) epilepsy; 12.07 years (2.82) cerebral palsy
Dardas & Ahmad, 2015 [23] (Jordan)	Cross-sectional	184	Autistic disorder	Parents of children under the age of 12 years with a clinical diagnosis of autistic disorder and could read and write in Arabic.	Mother, 62%	37 years (SD = 7.6, range from 21 to 69)	6.3 years (SD = 3, range = 2–12)
Grey et al., 2011 [63] (USA)	Randomized controlled trial	123 (coping skills training, *n* = 75; group education, *n* = 48)	Type 1 diabetes	Parent of a child who had been diagnosed with Type 1 diabetes for at least 6 months and between the ages of 1–12 years.	95% mothers, 3% fathers; 2% guardians (female)	37.3 years (SD = 5.6, range = 26–51)	8.1 (2.9) coping skills training; 7.9 (2.8) group education
Guillamon et al., 2013 [48] (Spain)	Cross-sectional	62	Cerebral palsy	Father or mother of a child with cerebral palsy (aged less than 18 years) and was the main caregiver.	Mother, 88.7%	40.95 years (SD = 0.79, range = 29.53)	7.69 years (SD = 0.19, range = 1–17)
Hamama-Raz & Hamama, 2015 [60] (Israel)	Cross-sectional	48	Epilepsy	Parents of children between 6 and 19 years of age, with Hebrew speaking ability, with only minor learning difficulties, with 1–4 seizures a year, and with the absence of other chronic illnesses.	Mother, 85.42%	42.90 years (SD = 6.20)	13.71 years (SD = 3.02, range = 8–18)
Khanna et al., 2011 [52] (USA)	Cross-sectional	304	Autism	Primary caregivers of children with autism aged less than or equal to 18 years of age and had no more than one child diagnosed with autism.	Female, 93.1%; relationship not stated	38.9 years (SD = 8.0)	7% < 5 years of age; 44.1% 5-less than 10 years; 41.4% 10-less than 15 years; 6.6% 15–18 years of age
Khanna et al., 2013 [59] (USA)	Cross-sectional	316	Autism	Primary caregivers of children with autism who are aged 18 years or younger and have only one child with autism.	Mother, 91.5%	18–44 years, 69%; 45–64 years, 30.1%	< 5 years, 16.8%; 5–10 years, 46.5%; 11–18 years, 36.1%
Motaharian et al., 2015 [53] (Iran)	Cross-sectional	49	Hemophilia	Primary caregiver (primary responsibility of providing care to child) of a child (less than 18 years of age) with hemophilia.	Male, 71.4% (relation not stated)	40 years or younger (44.9%); Older than 40 years (55.1%)	Twelve years or younger (46.9%); Older than 12 years (53.1%
Raina et al., 2005 [54] (Canada)	Cross-sectional	468	Cerebral palsy	Primary caregiver who had a child who participated in the Ontario Motor Growth (OMG) study (explored patterns of gross motor development in children with cerebral palsy), lived with the child, and resided in Ontario.	Mother, 89.7%	40.3 years (SD = 6.72)	10.6 years (SD = 2.69)
Streisand et al., 2010 [57] (USA)	Cross-sectional	278	Diabetes (type 1 or type 2)	Parent or guardian who was the most informed about the child’s health, child who was less than 18 years of age and was diagnosed with diabetes by a physician.	Mother (biological, step, foster, or adoptive), 100%	Not reported	12.1 years (SD = 4.3, range = < 1–17)
Tseng et al., 2016 [49] (Taiwan)	Cross-sectional	167	Cerebral palsy	Primary caregiver of a child with cerebral palsy aged 4 to 12 years, diagnosed by a pediatrician, pediatric neurologist, or physiatrist, and without an additional diagnosis of a neurodegenerative disease or psychiatric illness.	Mother, 82.0%; Father, 13.8%	40.24 years (SD = 5.43)	Range 4–12 years

### Instruments used to measure key constructs

#### Measurement of quality of life

A variety of instruments were used to measure QoL (Table 3). The World Health Organization Quality of Life Assessment Questionnaire (WHOQOL-BREF) [47] was used in 4 studies [23, 24, 48, 49]. The WHOQOL-BREF is comprised of 26 items and measures the following domains: physical health, psychological health, social relationships, and environment. Of the studies using this QoL measurement instrument, one did not include the environment sub scale but provided the other four sub scale scores [24], while one study used the overall QoL summary score only [23]. Two studies reported all sub scale scores [48, 49]; one of these [48] supplemented the psychological health subscales from the WHOQOL-BREF with measures of depression (the Beck depression inventory, BDI [50]) and anxiety (the State Trait Anxiety Inventory, STAI [51]).

**Table 3:** Self-administered measurement tools used to examine coping strategies, quality of life, and caregiving complexity

Measurement tool	Description of the tool	Reviewed studies using this tool
*Quality of life instruments*		
World Health Organization Quality of Life Assessment Questionnaire (WHOQOL-BREF) [47]	Latent variable composed of four subscales (physical, environmental, psychological, and social relationships) for a total of 26 items; higher score indicates better quality of life. Sub scale scores or a total overall scale can be computed.	[23, 24, 48, 49]
Medical Outcomes Study Short Form 36 Health Survey (SF-36) [55]	Generic measure of health concepts related to functional status and well-being; comprised of 8 domains and provides summary score for physical and psychological well-being.	[54]
Medical Outcomes Study Short-Form Health Survey version 2 (SF-12v2) [56]	Comprised of 12 items and 8 health concept subscales, also gives a summary score for physical and mental health status. Physical component sumamry (PCS) is made up of the physical functioning, role physical, bodily pain and general health subscales whereas the mental component (MCS) is comprised of the vitality, social functioning, role emotional, and mental health subscales.	[52, 53]
EuroQoL five-dimensional (EQ-5D) questionnaire [58]	Consists of five health profile domains: mobility, self-care, usual activities, pain/discomfort and anxiety/depression, each domain is assessed using a single item. A visual analogue scale is also used and ask caregivers to rate their current health status (range from 0 to 100).	[59]
Quality of Life in Pediatric Epilepsy Scale – Parent Form [61]	Comprised of four sub scales: psychological, physiological, functional, and social, with higher score indicating lower QoL.	[60]
Parents Diabetes Quality of Life Questionnaire [62]	Assesses parents’ perceptions of the impact of diabetes treatment on their general satisfaction with life; comprised of three subscales: diabetes life satisfaction, disease impact, and disease-related worries.	[63]
*Coping instruments*		
Coping Health Inventory for Parents (CHIP) [37]	Comprised of 3 subscales: 1) maintaining family integration, cooperation and optimistic definition of the situation; 2) maintaining social support, self-esteem and psychological stability; 3) understanding the healthcare situation through communication with other parents and consultation with the healthcare team.	[48, 54]
Brief COPE Inventory [33]	Comprised of 14 sub scales with two items each; broadly captures coping methods. Sub scales can be summarized into adaptive or maladaptive coping strategies.	[24, 52, 59]
Revised Ways of Coping Checklist [31]	Consists of 66 items separated into eight different cognitive and behavioural strategies (sub scales) used to cope with stressful encounters: confrontive coping, distancing, self-controlling, seeking social support, accepting responsibility, escape avoidance, planful problem solving, and positive reappraisal.	[23]
Calsbeek Coping Inventory for Stressful Situations (CISS) [65]	Comprised of 21 items in three domains: problem-oriented, emotion-oriented, and avoidance-oriented coping strategies.	[53]
Perceived Ability to Cope with Trauma Scale (PACT) [66]	Comprised of two sub scales: emotional processing and forward focus, single score used.	[60]
Issues in Coping with IDDM-Parent Scale [67]	Measures parents’ issues in coping with their child’s diabetes; Consists of two sub scales: how difficult and how upsetting parents find it to cope with issues related to management of child’s Type 1 Diabetes.	[63]
Family Coping Patterns Questionnaire (FCPQ) [68]	Comprised of 34 items in 3 sub scales: avoidance-oriented coping, cognitive appraisal-oriented coping, and task-oriented coping strategies	[49]
*Caregiving complexity instruments*		
Revised Burden Measure [74]	Latent variable composed of three subscales: Relationship Burden, Objective Burden, Subjective Burden.	[24]
Revised Scale for Caregiving Self-Efficacy [75]	Consists of three domains: obtaining respite, responding to disruptive patient behaviours, and controlling upsetting thoughts. Sub scale and overall scores.	[48]
Caregiver Strain Questionnaire [76]	Consists of three domains: objective strain, subjective internalized strain, and subjective externalized strain, an overall burden score is used.	[52, 59]
Pediatric Evaluation of Disability Inventory (PEDI) [77]	Measures amount of caregiver assistance provided to a child during basic functional activities of daily living.	[49, 54]

Three studies [52–54] used a version of the Medical Outcomes Study Short-Form Health Survey, one using the Short Form 36 (SF-36) [55] and two using the Short Form 12 (SF-12) [56]. Both versions provide subscale scores for eight health concepts as well as summary scores for physical and mental health.

Streisand et al. [57] used a single psychological well-being item and a single physical well-being item to measure the QoL of parents of children with diabetes. Each item asked respondents to rate their mental or physical health on a scale from 1 to 5.

One study used the EuroQoL five-dimensional questionnaire (EQ-5D) [58] to measure parental QoL [59]. The EQ-5D consists of five health profile domains: mobility, self-care, usual activities, pain/discomfort, and anxiety/depression. Each domain is assessed using a single item.

Hamama-Raz and Hamama [60] used the Quality of Life in Pediatric Epilepsy Scale – Parent Form [61]. The instrument is comprised of 16 items and four subscales: physiological, functional, psychological, and social QoL. An overall QoL score is also computed.

The parents Diabetes Quality of Life Questionnaire [62] was used to assess parents’ QoL in the study by Grey et al. [63]. The instrument consists of 47 items and three subscales: diabetes life satisfaction, disease impact, and disease-related worries. Only the disease impact subscale was reported.

#### Measurement of coping

Many self-reported instruments were used to measure caregiver coping strategies (Table 3). Two studies [48, 54] used the Coping Health Inventory for Parents (CHIP) [37]. The CHIP consists of 45 items and three subscales: 1) maintaining family integration, cooperation and optimistic definition of the situation, 2) maintaining social support, self-esteem, and 3) psychological stability, and understanding the healthcare situation through communication with other parents and consultation with the healthcare team.

Three studies [24, 52, 59] used the Brief Coping Orientation to Problem Experiences (brief-COPE) [33]. This tool contains 28 items organized into 14 subscales: active coping, planning, positive reframing, acceptance, humor, religion, use of emotional support, use of instrumental support, self-distraction, denial, venting, substance use, behavioural disengagement, and self-blame. The 14 subscales can further be classified into two categories, adaptive and maladaptive coping [64]. Two studies [52, 59] reported adaptive and maladaptive coping scores while Carona et al. [24] used only the behavioural disengagement subscale of the brief COPE.

Dardas and Ahmad [23] used the revised Ways of Coping Checklist [31] which contains 66 items and eight subscales representing cognitive and behavioural coping strategies: confrontive coping, distancing, self-controlling, seeking social support, accepting responsibility, escape avoidance, planful problem solving, and positive reappraisal.

Streisand et al. [57] used a single coping item from the National Survey of Children’s Health (NSCH) to assess parental coping strategies. The parent coping item asked respondents to rate how well they felt they were coping with the day-to-day demands of parenthood and raising children. Questions were measured on a four-point Likert scale.

The Coping Inventory for Stressful Situations-21 (CISS-21) [65] was used to measure parental coping strategies in one included study [53]. The CISS-21 contains 21 items and is made up of three subscales: task/problem-oriented, emotion-oriented, and avoidance-oriented coping.

Hamama-Raz and Hamama [60] used the 20 item Perceived Ability to Cope with Trauma Scale (PACT) [66] to measure coping strategies. The PACT consists of two subscales, emotional processing and a forward focus, and creates a single flexibility score which represents a balance between the two subscales.

In one study, the Issues in Coping with IDDM-Parent Scale [67] was used to measure coping strategies of parents of children with type 1 diabetes [63]. The measurement instrument assesses, in two sub scales, how difficult and how upsetting parents find coping with issues related to management of their child’s type 1 diabetes. In the included study [63], the mean of the two scales was used as an overall coping score.

Tseng and colleagues [49] used the Family Coping Patterns Questionnaire (FCPQ) [68] to measure the coping strategies used by caregivers of children with cerebral palsy. The FCPQ has 34 items and consists of 3 subscales that measure the use and perceived helpfulness of avoidance-oriented, appraisal-oriented, and task-oriented coping strategies.

### Quality assessment

The quality of included studies was generally fair or good based on the NIH quality assessment tool for observational cohort and cross-sectional studies (Table 4). All studies clearly defined the research question or objective and the study population of interest. Fifty percent participation rate was achieved in 4 studies, was not achieved in 3 studies and was not reported in 4 studies. All but one study [24] recruited participants from the same or similar populations; this study included children diagnosed with cerebral palsy and epilepsy and recruited participants using different methods. A majority (9/11) of studies did not report sample size justification, power description, or variance and effect estimates. All cross-sectional studies (10/11) did not assess the exposure of interest prior to outcome measurement as assessments occurred at the same time; therefore these studies also received a “no” for sufficient timeframe to see an association between exposure and outcome and multiple exposure measurements. All studies clearly defined and consistently implemented exposure and outcome measures however one study [57] used single-item questions, rather than validated instruments, to measure coping and QoL. Blinding of outcome assessors did not pertain to the cross-sectional studies and was not reported in the RCT. Similarly, loss to follow-up was not applicable to the cross-sectional studies and was less than 20% in the RCT. Eight studies considered potential confounding variables in the relationship between coping strategies and QoL.

**Table 4:** Quality assessment for included studies: NIH Quality Assessment Tool for Observational Cohort and Cross-Sectional Studies

Quality Assessment Criteria	Study										
	Carona et al. (2014) [24]	Dardas &Ahmad (2015) [23]	Grey et al. (2011) [63]	Guillamon et al. (2013) [48]	Hamama-Raz & Hamama (2015) [60]	Khanna et al. (2011) [52]	Khanna et al. (2013) [59]	Motaharian et al. (2015) [53]	Raina et al. (2005) [54]	Streisand et al. (2010) [57]	Tseng et al. (2016) [49]
Was the research question or objective in this paper clearly stated?	✓	✓	✓	✓	✓	✓	✓	✓	✓	✓	✓
Was the study population clearly specified and defined?	✓	✓	✓	✓	✓	✓	✓	✓	✓	✓	✓
Was the participation rate of eligible persons at least 50%?	✗	NR	✓	NR	✗	✗	NR	✓	✓	✓	NR
Were all the subjects selected or recruited from the same or similar populations (including the same time period)? Were inclusion and exclusion criteria for being in the study prespecified and applied uniformly to all participants?	✗	✓	✓	✓	✓	✓	✓	✓	✓	✓	✓
Was a sample size justification, power description, or variance and effect estimates provided?	✗	✓	✗	✗	✓	✗	✗	✗	✗	✗	✗
For the analyses in this paper, were the exposure(s) of interest measured prior to the outcome(s) being measured?	✗	✗	✓	✗	✗	✗	✗	✗	✗	✗	✗
Was the timeframe sufficient so that one could reasonably expect to see an association between exposure and outcome if it existed?	✗	✗	✓	✗	✗	✗	✗	✗	✗	✗	✗
For exposures that can vary in amount or level, did the study examine different levels of the exposure as related to the outcome (e.g., categories of exposure, or exposure measured as continuous variable)?	N/A	N/A	N/A	N/A	N/A	N/A	N/A	N/A	N/A	N/A	N/A
Were the exposure measures (independent variables) clearly defined, valid, reliable, and implemented consistently across all study participants?	✓	✓	✓	✓	✓	✓	✓	✓	✓	✗	✓
Was the exposure(s) assessed more than once over time?	N/A	N/A	✓	N/A	N/A	N/A	N/A	N/A	N/A	N/A	N/A
Were the outcome measures (dependent variables) clearly defined, valid, reliable, and implemented consistently across all study participants?	✓	✓	✓	✓	✓	✓	✓	✓	✓	✗	✓
Were the outcome assessors blinded to the exposure status of participants?	N/A	N/A	NR	N/A	N/A	N/A	N/A	N/A	N/A	N/A	N/A
Was loss to follow-up after baseline 20% or less?	N/A	N/A	✓	N/A	N/A	N/A	N/A	N/A	N/A	N/A	N/A
Were key potential confounding variables measured and adjusted statistically for their impact on the relationship between exposure(s) and outcome(s)?	✗	✗	✓	✓	✓	✓	✓	✗	✓	✓	✓
Overall Quality	Good	Fair	Good	Good	Good	Good	Good	Poor	Good	Poor	Good

*NR* Not reported, *N/A* Not applicable

The Cochrane risk of bias tool was also used to assess the quality of the one included RCT. A high or unclear risk of bias was determined for several reasons. The authors did not mention how randomization of study participants occurred. There was no mention of how blinding (if any) of participants to intervention group was done. In addition, blinding of outcome assessors was not reported.

### Findings regarding the association between caregiving complexity and quality of life

Six studies analyzed the relationship of child disease severity and/or some form of caregiving complexity with QoL in caregivers of children with chronic illness [24, 48, 49, 52, 54, 59] (Table 5). The results were mixed but generally suggestive that greater caregiving complexity or needs may be associated with poorer caregiver well-being.

**Table 5:** Variables of interest, approach to analysis, and main findings of included studies

Author, year	Variables of interest	General approach to analysis	Main findings
Carona et al., 2014 [24]	Coping: behavourial disengagement (avoidant, emotion-focused strategy) QoL: overall QoL Caregiving complexity: caregiving burden measured	Structural equation modelling	(1) Caregiving burden directly and negatively predicted parents’ QoL (2) Behavioural disengagement directly and negatively predicted parents’ QoL (3) Caregiving burden had a significant indirect effect on parent’s QoL via behavioural disengagement coping. (4) Coping had a mediating role in the association between caregiving burden and quality of life.
Dardas & Ahmad, 2015 [23]	Coping: eight sub scale measurements QoL: overall QoL	Bivariate and multivariable regression	(1) Escape avoidance and accepting responsibility coping strategies were significantly and inversely associated with QoL. (2) Accepting responsibility was found to mediate the association between stress and QoL. (3) Escape avoidance and seeking social support were found to moderate the relationship between stress and QoL.
Grey et al., 2011 [63]	Coping: issues in coping measurement (higher score = coping is more upsetting and difficult) QoL: Disease impact on general life satisfaction (higher score = greater negative impact)	Correlation	(1) While controlling for baseline coping, change in coping at 3 months was not significantly correlated with change in quality of life at 3, 6, or 12 months.
Guillamon et al., 2013 [48]	Coping: sub scale measurements of integration, social support, and understanding QoL: sub scale measurements of physical, social relationships, environment (considered QoL), and mental health, BDI, and STAI-Trait (considered to be mental health). Caregiving complexity: self-efficacy measured	Multivariable regression	(1) Coping strategies did not significantly predict any quality of life or mental health indicators. (2) Caregiving self-efficacy was a significant, positive predictor of the quality of life environment and mental health sub scales, and significantly predicted caregiver anxiety.
Hamama-Raz & Hamama, 2015 [60]	Coping: flexibility measurement QoL: sub scale measurements (physical, psychological, social, and functional) as well as overall QoL (higher score = lower QoL)	Correlation and multivariable regression	(1) Flexibility coping was significantly and inversely correlated with each QoL sub scale as well as overall QoL. (2) Flexibility coping was significantly and negatively associated with psychological, functional, and overall QoL (i.e., greater use of flexibility coping was associated with a decreased negative effect of QoL).
Khanna et al., 2011 [52]	Coping: adaptive and maladaptive sub scale measurements QoL: physical and psychological sub scale measurements Caregiving complexity: caregiving burden measured Disease severity: care recipient functional status	Multivariable regression and structural equation modelling	(1) Care recipient functional status, maladaptive coping, and caregiver burden were significantly and negatively associated with psychological QoL. (2) Maladaptive coping had a direct negative effect on psychological QoL. (3) Adaptive and maladaptive coping had a direct positive effect on caregiver burden which in turn had a direct negative effect on psychological QoL.
Khanna et al., 2013 [59]	Coping: adaptive and maladaptive sub scale measurements QoL: overall health-related QoL Caregiving complexity: three sub scale measurements- objective strain, subjective internalized strain, and subjective externalized strain Disease severity: parent-reported measurement of social interaction, communication, and restricted and repetitive behavior (overall score given)	Correlation and multivariable regression	(1) Objective and subjective internalized strain were significantly and inversely correlated with QoL. (2) Maladaptive coping was significantly and inversely correlated with QoL. (3) Objective strain was a significantly and negatively associated with QoL.
Motaharian et al., 2015 [53]	Coping: three sub scale measurements given (problem-, emotion-, and avoidance-oriented) QoL: overall QoL	Correlation and multivariable regression	(1) Emotion- and avoidance-oriented coping was significantly and inversely correlated with QoL. (2) Emotion- and avoidance-oriented coping were significantly and negatively associated with QoL in the regression model.
Raina et al., 2005 [54]	Coping: stress management QoL: sub scale measurements of physical and psychological QoL Caregiving complexity: caregiving demand	Structural equation modelling	(1) Stress management (coping) had a direct positive effect on caregiver psychological health. (2) Caregiving demand was directly and positively associated with physical and psychological health QoL of caregivers (greater score = less demand).
Streisand et al., 2010 [57]	Coping: single item, respondents asked how well they felt they were coping with the day-to-day demands of parenthood and raising children. QoL: Parent physical and psychological well-being measured using single item.	Bivariate associations and multivariable regression	(1) Coping was significantly associated with psychological and physical QoL in bivariate and multivariate models.
Tseng et al., 2016 [49]	Coping: three sub scale measurements given QoL: sub scale measurements of physical, psychological, social relationships, and environment Caregiving complexity: factor analysis of items from two sub scales of the Chinese version of the Pediatric Evaluation of Disability and Inventory (measuring Functional Skills and Caregiver Assistance) Disease severity: scales that measured gross motor impairment severity and fine motor impairment severity	Multivariable regression	(1) Greater use of avoidance-oriented coping positively associated with all QoL domains; perceived helpfulness or use of cognitive appraisal-oriented coping associated with higher psychological and social QoL, respectively; perceived helpfulness of task-oriented coping associated with higher environment QoL (2) Degree of child fine motor impairment associated with physical, social, and environment domains of QoL but not with psychological QoL (3) Caregiving complexity (functional skills and need for caregiver assistance) not included in final (stepwise) multivariable models

Specifically, the following aspects of disease severity or caregiving complexity were addressed: overall disease severity [52, 59] or motor impairment severity [49]; caregiving ‘burden’ or ‘objective strain’ [24, 52, 59]; caregiving demands or need for caregiver assistance [49, 54]; and caregiving self-efficacy [48]. With respect to *child disease severity*, one study found no significant association between disease severity and caregiver QoL [59], while a second found that disease severity was significantly and negatively associated with caregiver psychological QoL [52] and a third study found that increased child motor impairment was associated with some but not all domains of caregiver QoL [49]. With respect to *objective strain (a subscale of the caregiver strain questionnaire) and/or caregiving ‘burden’*, two studies found these variables to be significantly and negatively associated with overall caregiver well-being [24, 59] while another study found that caregiving ‘burden’ was significantly and negatively associated with psychological QoL specifically [52]. With respect to *caregiving demands or need for caregiver assistance*, one study found that having fewer demands was significantly, directly, and positively associated with both psychological and physical QoL [54] while in a second study measures of need for caregiver assistance were not significant in final multivariable models for any QoL domains [49]. Finally, Guillamon et al. [48] reported on *caregiving self-efficacy*, which was significantly and positively associated with environmental and psychological health sub scales of QoL and was also a significant negative predictor of caregiver anxiety.

### Findings regarding the association between coping strategies and quality of life

Across the 11 studies included in the review, 8 found at least some evidence that there were significant associations between coping strategies used by caregivers and caregiver QoL. While this differed according to types of coping and components of QoL, the clearest evidence was related to psychological QoL.

#### Coping and global QoL

Six studies reported overall QoL in association with coping strategies [23, 24, 53, 59, 60, 63]. Two studies found no significant association between the two variables [59, 63], while the remaining four found coping strategies to be a significant predictor of caregiver QoL [23, 24, 53, 60]. While poor coping strategies (e.g., behavioural disengagement, escape avoidance, emotion-oriented) were negatively associated with QoL [23, 24, 53], in some studies strategies considered to be adaptive (e.g., problem-oriented, accepting responsibility) were also negatively associated with QoL [23, 53]. Greater use of flexibility as a coping strategy was found in one study to be significantly associated with better QoL [60].

#### Coping and physical aspects of QoL

Coping strategies were not significantly associated with physical health in 4 studies [48, 52, 54, 60]. However, Streisand et al. [57] found that a single coping item was significantly and positively associated with both psychological and physical well-being (both of which were also measured using a single item); and Tseng et all [49] found that greater use of avoidance-oriented coping was positively associated with physical QoL.

#### Coping and psychological QoL

Four studies found that coping strategies were significantly associated with caregiver psychological QoL [49, 52, 54, 60] while one study found no significant association [48]. Specifically, Raina et al. [54] identified that greater use of stress management was positively and directly associated with psychological health; Khanna et al. [52] found that greater use of maladaptive coping strategies was directly and negatively associated with caregiver psychological health; Tseng and colleagues found that use of avoidance-oriented coping and perceived helpfulness of cognitive appraisal-oriented coping strategies were both positively associated with psychological QoL [49]; and Hamama-Raz and Hamama [60] found that caregivers who used flexibility as a coping strategy had better psychological, functional, and overall QoL.

### Findings regarding the role of coping strategies as a mediator or moderator of the association between caregiving complexity and quality of life

Two studies, both cross-sectional, also examined coping strategies as a potential mediator of the association between disease severity or caregiving complexity and QoL [24, 52]. Carona et al. [24] found that behavioural disengagement coping among caregivers, which is characterized by “reducing one’s effort to deal with the stressor or even quitting the attempts to achieve goals with which the stressor is interfering” (p. 321) mediated the association between caregiving complexity and QoL, helping to explain how caregiving complexity may influence parental QoL. Specifically, their findings suggested that among parents of children with cerebral palsy or epilepsy who experienced increased caregiving complexity, this additional complexity may have impaired their ability to cope and, specifically, elicited greater use of behavioural disengagement coping strategies. These coping strategies were in turn associated with poorer QoL [24].

In contrast, Khanna et al. [52] found that maladaptive coping strategies, defined as “emotion-focused strategies that aim to regulate the distress associated with the problem” [36], did not mediate the association between disease severity and caregiver psychological QoL in their multivariable model, nor was this a reported pathway in their final structural equation model. In addition, in their study, both maladaptive and adaptive coping strategies, the latter defined as “problem-focused coping strategies used to directly address the problem causing distress” [36] had a direct positive effect on caregiving ‘burden’ which, in turn, had a direct negative effect on psychological QoL in the structural equation model (maladaptive coping also had a significant direct negative effect on psychological QoL in the final model) [52].

None of the reviewed studies examined coping strategies as a potential moderator (effect modifier) of the association between disease severity or caregiving complexity and QoL.

## Discussion

This review sought to better understand the association between coping strategies and QoL among caregivers of children with chronic illness, and, in particular, the potential role of coping strategies as a mediator of the association between caregiving complexity and QoL or as a moderator of that association. To our knowledge, this is the first review to consider these associations in this population.

Our findings support an association between coping strategies and psychological aspects of QoL. Adaptive coping strategies were positively associated with psychological aspects of QoL in several of the reviewed studies, although differences in how coping was measured made it challenging to categorize strategies definitively. Conversely, maladaptive coping strategies were negatively associated with psychological aspects of QoL in one study [52]. These findings are further supported by a number of studies of families of children with intellectual disability (ineligible for our review as they frequently did not meet our inclusion criteria with respect to diseases requiring pediatric specialist interventions), which have reported improved caregiver well-being associated with problem-focused coping strategies [69, 70], while emotion-focused strategies may be associated with poorer caregiver well-being [71].

By contrast, both adaptive and maladaptive strategies were associated with decreased overall QoL (psychological and physical health combined) in four studies. This may indicate that caregivers who use more coping strategies (both adaptive and maladaptive) may have a greater need to cope – i.e., they have more stress that requires coping. This is consistent with findings from a review by Cousino and Hazen [72] that analyzed parenting stress among caregivers of children with chronic illness and it underscores the need for prospective studies that can measure stressors (such as caregiving complexity), coping responses, and QoL.

We identified only two studies that examined the role of coping strategies as a mediator (intermediate variable) of the association between caregiving complexity and QoL, both cross-sectional. One of these two studies found behavioural disengagement coping to mediate the association between caregiving ‘burden’ and QoL [24]. The second study did not find coping (adaptive or maladaptive strategies) to be a potential mediator in the association between disease severity and QoL using hierarchical regression [52]. These findings suggest that while coping strategies could play an important role in mediating the association between caregiving complexity and psychological aspects of QoL, prospective research is needed to clarify the nature of these relationships.

We did not find any studies that examined the potential role of coping as a moderator (i.e., effect modifier) of the relationship between caregiving complexity and caregiver QoL. However, previous studies of parents of children with chronic illness or disability have examined coping as a possible moderator in the association between *caregiving stress and parental QoL* [23] or between *caregiving complexity and parental stress* [38]. Specifically, Dardas and Ahmad found that the coping strategies of seeking social support and escape-avoidance acted as buffers in the association between parental stress and parental QoL [23]. Lyons and colleagues found that both emotion-oriented coping and a form of avoidance-oriented coping (distraction) were associated with lower scores on measures of stress among those parents of children with more severe symptoms [38]. These findings suggest that it may be worthwhile for future research to also investigate whether coping strategies moderate the association between caregiving complexity and parental QoL. Understanding whether specific coping strategies have the potential to mitigate the stress associated with highly complex caregiving is important for identifying how best to support families of children with chronic illness.

This review identified just 11 studies that met our eligibility criteria, only two of which specifically examined the potential mediating role of coping in the association between caregiving complexity and caregiver QoL. In addition, although we identified some consistency in positive associations between use of positive or adaptive coping strategies and psychological QoL among parents of children with chronic illness, the findings of the studies we reviewed were challenging to synthesize due to great diversity in how disease severity, caregiving complexity, and coping strategies were measured. In particular, our findings corroborated that there is a lack of conceptual clarity about parental coping: the 7 different instruments used to measure coping across the 11 studies did not consistently define coping or categorize it in terms of its dimensions. Future studies that focus on coping and QoL in this population should seek to better understand the conceptual underpinnings of this construct.

In addition to this challenge related to conceptual and measurement clarity/consistency, a second important issue across the studies we reviewed was the lack of prospective research: all but one of the studies we reviewed used cross-sectional study designs, making it difficult to determine if caregivers who use adaptive coping mechanisms are healthier as a result of their improved coping ability or if caregivers with greater QoL are able better able to respond to their environment due to their improved health. Similarly, one of the analyses reported in the study by Khanna et al. [52] positioned coping as having an influence on caregiving complexity (rather than the reverse), which in turn influenced QoL, further demonstrating the need for prospective studies to clarify the inter-relationships between the complexity of caregiving needs, strategies for coping with such needs, and caregiver well-being.

Resolving these remaining research gaps has implications for the development of interventions to improve caregiver well-being. A Cochrane review of psychological interventions for parents of children and adolescents with chronic illness found little evidence regarding the efficacy of psychological therapies for parents on several outcome domains of functioning, such as parental mental health [1]. However, that review highlighted that parent mental health and adaptive behavior can improve when parents participate in problem solving therapy. Similarly, in studies of children with diabetes, promising results have been found regarding caregivers’ response to interventions aimed at providing skills to manage uncertainty [73], coping skills training, and education sessions [63]. These studies show the potential benefits that interventions such as group-based education or coping training sessions may have on caregivers of chronically ill children and highlight the importance of continuing to study how coping affects well-being.

Our review has important strengths, including its synthesis, for the first time, of studies reporting quantitative associations between caregiving complexity, coping, and QoL in parents of children with chronic illness; and our focus on assessing study quality and identifying important research gaps. However, findings from this review must be interpreted in light of its limitations. First, while attempts were made to include all relevant research, our search strategies were limited to English language articles only. Secondly, the inclusion criteria used in this review needed to be strict in order to develop a search strategy that was feasible given the non-specific language used to describe coping and well-being and the large literature on chronic pediatric illness in general. This narrowed the scope of the included articles, for example, in terms of the illnesses studied and the methods of identifying articles describing those illnesses. For example, searching illness etiology using only MeSH headings was an important limitation. We focused on illnesses that would be very likely to have an early, long-lasting, and important impact on caregivers in order to have the best chance of uncovering associations between caregiving complexity, coping, and QoL. However, our focus on diseases of genetic, metabolic, and neurologic etiology likely resulted in the exclusion of some papers describing conditions that would have an early and long-lasting caregiver impact but that fall outside those three etiologies (e.g., within the fields of hematology/oncology, cardiology, pulmonology, or gastroenterology).

The review was also restricted to studies that included quantitative measures of caregiver coping and quality of life. Findings from qualitative studies are essential to understanding factors that contribute to the well-being of caregivers who are often under studied in the literature but whose experiences are unique and important to consider. However, for our purposes, we were specifically interested in quantitative estimates of association between coping and QoL and, particularly, studies that had examined coping as a potential mediating variable (to explain the potential association between caregiving complexity and QoL) or moderating variable (potentially altering the association between caregiving complexity and QoL). Lastly, our conclusions are limited by the inherent limitations of the included articles, which, as described, include a lack of consistency in defining and measuring key constructs, and the lack of prospective studies to clarify the temporal order of the associations identified.

## Conclusions

Findings from our review support the hypothesis that positive or adaptive coping strategies may be positively associated with psychological QoL among caregivers of children with chronic illness. If future studies using prospective designs provide further support for a causal relationship between coping and QoL, our results highlight the potential value of interventions targeted at caregiver coping processes to improve the well-being of caregivers and, in turn, their children. In addition to prospective studies, our findings highlight the need for future research to investigate whether coping strategies may moderate the association between caregiving complexity and parental QoL, and for clarification of the concepts and measurement of coping in this body of literature.

## Additional file

Additional file 1: Search strategies used in electronic databases. (DOCX 17 kb)

## Data Availability

All data generated or analysed during this study are included in this published article.

## Abbreviations

ASD: Autism Spectrum Disorders
BDI: the Beck depression inventory
brief-COPE: Brief Coping Orientation to Problem Experiences
CHIP: Coping Health Inventory for Parents
CISS-21: Coping Inventory for Stressful Situations-21
EQ-5D: EuroQoL five-dimensional questionnaire
MeSH: Medical Subject Headings
NSCH: National Survey of Children’s Health
PACT: Perceived Ability to Cope with Trauma Scale
PRISMA: Preferred Reporting Items for Systematic Reviews and Meta-Analyses
PRISMA-P: Preferred Reporting Items for Systematic Reviews and Meta-Analyses- Protocol
QoL: Quality of life
RCT: Randomized controlled trial
SF-12: Short Form 12 health survey
SF-36: Short Form 36 health survey
STAI: State Trait Anxiety Inventory
WHO: World Health Organization
WHOQOL-BREF: World Health Organization Quality of Life Assessment Questionnaire
