# Supplementary material for: A systematic review of the association between coping strategies and quality of life among caregivers of children with chronic illness and/or disability
Source: BMC Pediatr. 2019 Jul 1;19:215. doi: 10.1186/s12887-019-1587-3 (PMC6600882; doi:10.1186/s12887-019-1587-3)
Supplement: Supplementary file 1 — Search strategies used in electronic databases. (DOCX 17 kb) [file 12887_2019_1587_MOESM1_ESM.docx]

Records identified by applying search strategy to electronic databases

(n= 3,568)

Duplicate records identified by software and removed (n= 950)

Records screened on basis of title and abstract

(n= 2,618)

Duplicate records manually identified and removed (n= 16)

Failure to meet inclusion criteria

(n= 2,413)

Records eligible for full-text screen

(n= 189)

Additional records identified through other sources (n=3)

Duplicate records manually identified and removed (n= 4)

Exclusion reasons (not mutually exclusive):

Abstract only (n= 23)

Coping not measured in caregiver (n= 88)

QoL not measured in caregiver (n= 101)

Non-English (n=1)

Study design ineligible (n= 13)

Pediatric illness ineligible (n= 9)

Pediatric age exceeds 18 years (n= 37)

Total articles excluded (n= 177)

Studies included in the review

(n=11)

**Figure 1.** PRISMA flow diagram for the systematic review of the association between coping strategies and quality of life among caregivers of children with chronic illness and/or disability
